# Fertility Preserving Surgery Outcomes for Ovarian Malignancy: Data from a Tertiary Cancer Centre in Central London

**DOI:** 10.3390/jcm11113195

**Published:** 2022-06-03

**Authors:** Jonathan Gaughran, Hannah Rosen O’Sullivan, Tom Lyne, Ahmed Abdelbar, Mostafa Abdalla, Ahmad Sayasneh

**Affiliations:** 1 Women’s Health, Guy’s and St Thomas’ NHS Foundation Trust, Westminster Bridge Road, London SE1 7EH, UK; hannah.rosenosullivan@gstt.nhs.uk; 2 Faculty of Life Sciences & Medicine at Guy’s, The School of Life Course Sciences, King’s College London, London WC2R 2LS, UK; tom.lyne@kcl.ac.uk (T.L.); ahmad.sayasneh@gstt.nhs.uk (A.S.); 3 Department of Gynaecological Oncology, Guy’s and St Thomas’ NHS Foundation Trust, Westminster Bridge Road, London SE1 7EH, UK; ahmed.abdelbar@gstt.nhs.uk (A.A.); mostafa.abdalla@gstt.nhs.uk (M.A.)

**Keywords:** fertility, ovarian, cancer, borderline, reproduction, surgery, preservation, oncology, gynaecology

## Abstract

Fertility Sparing Surgery (FSS) appears to be a safe means of treating early-stage ovarian cancer based on relatively limited evidence. However, there is currently insufficient evidence to aid women in counselling about their potential fertility outcomes. The aim of this study was to assess the reproductive outcomes and prognosis of women who have undergone FSS for ovarian malignancy. Between 1 June 2008 and 1 June 2018, a retrospective review of a clinical database was conducted to identify all consecutive patients who underwent FSS in a central London gynaecological oncology centre. All patients with a histological diagnosis of ovarian malignancy (excluding borderline ovarian tumours) were eligible. All identified patients were then prospectively called into a follow up and asked to complete a questionnaire about their fertility outcomes. A total of 47 women underwent FSS; 36 were included in this study. The mean age was 30.3 years (95% Confidence Interval [CI]: 27.6 to 33.0 years). During the study period, 17/36 (47.2%) of the women had attempted to conceive following surgery, with a successful live birth rate of 52.9% (9/17). The mean time of recurrence was 125.3 months (95% CI: 106.5–144.1 months). The mean time to death was 139.5 months (95% CI: 124.3–154.8). The cancer grade, tumour stage and use of Assisted Reproductive Technology (ART) were the main factors significantly associated with the risk of recurrence and death. In conclusion, this study suggests that a large proportion of women will not attempt to conceive following FSS. For those who do attempt to conceive, the likelihood of achieving a live birth is high. However, careful counselling about the higher risk of recurrence and worse survival for women with high grade cancer, disease Stage > IA and potentially those who undergo ART is essential before contemplating FFS.

## 1. Introduction

Ovarian cancer is the sixth most common cancer in females in the UK, accounting for 4% of all new cancer diagnoses [1]. Cytoreductive surgery, with or without chemotherapy, including hysterectomy and bilateral salpingo-oophorectomy, is the standard surgical management for patients with advanced staging, as robust evidence demonstrates that the absence of residual disease after surgery is a strong predictor of survival [2]. However, every year in the UK 1315 women of child bearing age are diagnosed with ovarian cancer [1]. In 15–20% of such cases, the disease is isolated to one ovary which yields a five-year survival of 90% [3]. As such, surgical sterilisation may not be an acceptable treatment choice for this cohort [3]. Fertility sparing surgery (FSS) is defined as the removal of disease whilst preserving the potential to conceive [4]. While this most commonly describes a unilateral salpingo-oophorectomy (USO) with conservation of the uterus, advances in assisted reproductive techniques (ART) have meant that in cases where bilateral salpingo-oophorectomy (BSO) is indicated, conservation of the uterus alone may permit future implantation of a frozen or donated embryo [5]. One of the challenges in counselling patients considering FSS is the fact that most studies to date mix the various histological subtypes of ovarian cancers as well as borderline ovarian tumours (BOT). Regardless of this, the evidence to date suggests that FSS in carefully selected patients yields a comparable prognosis to radical surgery, and live birth rates of up to 67% are expected [6]. A recent systematic review by Canlorbe and colleagues concluded that FSS could safely be offered to women with epithelial and non-epithelial ovarian cancers < 1C3 [6]. Additionally, a prospective study of 105 women with epithelial ovarian cancer (EOC) undergoing FSS demonstrated no difference in survival compared to women who underwent radical surgery for tumours staged < 2A [7]. The need for tumour specific counselling has been highlighted by some studies demonstrating potential worse outcomes following FSS in patients with any tumour with a pathological grade > 2 or histological subtype of clear cell carcinomas [8]. However, another large systematic review found no difference in survival for all stage 1 EOC regardless of grade or histological subtype [9]. At present, limited data suggests that there is a higher relapse rate in patients opting for FSS, which must be borne in mind when counselling [7]. The aim of this study was to assess the reproductive outcomes for women who underwent FSS specifically for ovarian cancer in a tertiary referral gynaecology oncology unit over a 10-year period. Secondary aims were to review the rate of recurrence and death, the histological subtypes diagnosed, the miscarriage rate, the use of ART, complications associated with resultant pregnancies and the mode of delivery. 

## 2. Materials and Methods

This was a prospective questionnaire cohort study. Ethical approval was granted by the South East London Research & Ethics Committee (reference number 20/LO/1220). A clinical database listing all patients who underwent FSS between 1 June 2008 and 1 June 2018 in a central London University Hospital tertiary gynaecological oncology centre was first reviewed. This review was conducted in September 2021. Only cases with final histology confirming ovarian malignancy were included. BOT are a distinct subgroup of ovarian neoplasms described as having ‘low malignant potential’ which occur in a younger cohort [9,10,11]. As such, BOT were not included in this study. Any live patients aged 16 years or over at the time of the study were considered eligible. Potential participants were informed of the study either during their follow up clinic appointment or by telephone. A patient information leaflet was provided and questions about the study answered. If written consent was obtained when required to fill the questionnaire, eight questions were asked about attempts at conception; complications in pregnancy; mode of delivery; and disease recurrence (Appendix A). The hospital electronic patient record was accessed in order to obtain the following information: age at time of surgery; date of surgery; type of surgery; disease stage at diagnosis as per the International Federation of Gynaecology and Obstetrics (FIGO) classification (2018), and tumour histology as per the World Health Organisation (WHO) criteria [12,13]. Data on recurrence and mortality was extracted from the hospital reporting systems (Clinical Manager©, version 2.0, iSOFT, Australia, 2007 and MOSAIQ^®^ oncology information system, Stockholm, Sweden, 2014).

### Statistical Analysis

MedCalc^®^ (MedCalc version 20.010, Ostend, Belgium, 2018) was used for statistical analysis [14]. The following information was calculated: mean age and gestation at diagnosis; number of women attempting to conceive and the mean number of pregnancies and children born in this cohort. Overall survival was calculated from the day of primary cytoreductive surgery until the day of death from any cause (event) or the last day of follow-up (censored). Losses to follow-up were regarded as censored observations. Progression-free survival was calculated from the date of cytoreductive surgery until the date of diagnosis of the first relapse, the date of death from any cause or the last day of follow-up, whichever happened earlier. Progression-free and overall survival were calculated using Kaplan-Meier analysis as well as the 95% confidence interval of the hazard ratio between treatment groups. A Chi-square test was performed to assess the relationship between recurrence or death rates and disease staging or tumour grade; association between recurrence and death after open versus laparoscopic surgery, and the association between recurrence and ovarian cystectomy versus oophorectomy and/or ART vs. natural pregnancies. A *p*-value < 0.05 was considered statistically significant.

## 3. Results

### 3.1. Demographics

FSS was undertaken in 47 cases. A total of 36 patients fulfilled the criteria for inclusion and agreed to fill the questionnaire. Of the remaining 11 patients, eight were recorded alive on electronic hospital and GP systems but uncontactable to give consent, and three declined to participate. Patient and tumour demogrpahics are displayed in Table 1. The mean age at time of surgery was 30.2 years (95% CI: 27.6 to 33.0 years). Ethnicity was as follows: Caucasian: 21/36 (58.3%); Black: 9/36 (25%); Middle Eastern: 3/36 (8.3%); South Asian: 2/36 (5.6%); South East Asian: 1/36 (2.8%). Comorbidities were documented in 3/36 (8.3%) at the time of surgery: Polycystic Ovarian Syndrome (PCOS) and successfully treated simple endometrial hyperplasia (1); asthma (1); obesity (1). Disease stage was: 1A (27/36 [75%]); 1C (5/36 [13.8%]); 2A (1/36 [2.8%]); 2C (1/36 [2.8%]) and 3C (2/36 [5.6%]). A USO was performed in 32/36 (88.9%) of cases and cystectomy in 4/36 (11.1%). Histology was available for all masses: EOC: 15/36 (41.7%) [seven mucinous cell adenocarcinomas, four clear cell carcinomas, two serous cell adenocarcinomas, one undifferentiated carcinoma, one poorly differentiated squamous cell carcinoma]; sex cord stromal tumours (SCST): 12/36 (33.3%) [11 adult granulosa cell tumours and 1 sclerosing stromal tumour]; germ cell ovarian tumours (GCOT): 9/36 (25%) [5 immature teratomas, 2 yolk sac germ cell tumours, 1 dysgerminoma, 1 mixed germ cell tumour). The tumours were classified as grade 1 in 22/36 (61%), grade 2 in 5/36 (14%) and grade 3 in 9/36 (25%).

### 3.2. Fertility Outcomes

A total of 17/36 women (47%) attempted to conceive following surgery. Of these 17, 10 (59%) had at least one live birth. There were a total of 17 live births in this cohort: five patients had one child, three patients had two children and two patients had three children. The mean number of children per patient was 1.7. In vitro fertilisation (IVF) was used in two out of 17 cases (12%). Delivery was by caesarean section in seven out of 17 cases (41%) [six elective, one emergency] and of these, none stated their previous surgery to be the reason for caesarean section. In two of 17 cases (17%), pregnancy related complications were identified: symphysis pubis dysfunction (1) and pre-eclampsia (1). Pre-term delivery occurred in three out of 17 (18%) pregnancies: two of which were due to medical induction of labour for twins; and the cause of the other was not found.

Of the seven out of 17 (41%) who did not achieve a live birth despite trying, six out of seven (86%) stated they had never had a positive pregnancy test. All but one (five out of six) were investigated for subfertility. The results were as follows: no abnormality detected: 2 (33.3%); endometriosis: 1 (16.7%); tubal factor: 1 (16.7%); anovulation presumed to be due to low BMI: 1 (16.7%).

**Figure 1:**
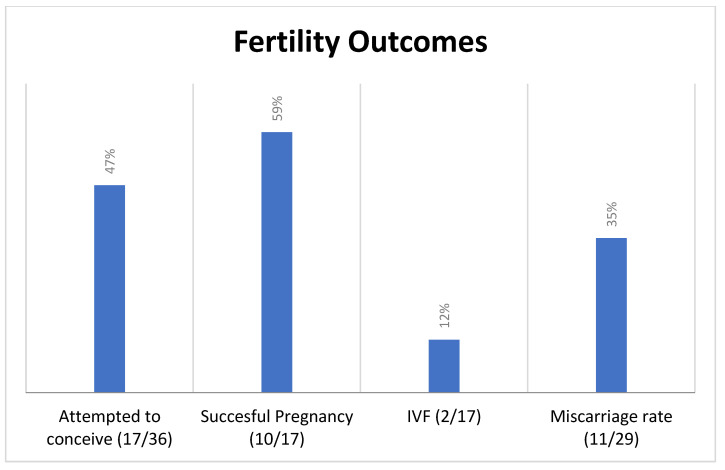
Fertility outcomes (%).

Participants reported a total of 11 pregnancy losses (defined as a positive pregnancy test followed by loss of pregnancy); all of which were in the first trimester. As there were a total of 29 positive pregnancy tests in the cohort (17 live births, 11 pregnancy losses and one termination), this equated to a miscarriage rate of 38%. Of the 11 pregnancy losses, none occurred in women over the age of 40 years. Investigations were performed for recurrent pregnancy loss in two patients in which no abnormality was detected. Of the 19/36 (53%) who had not attempted to become pregnant at the time of this study, their current age ranged from 18 to 42 (mean of 31 years). In four of 19 (21%) cases, FSS had occurred in the preceding five years, and a further four (21%) had died by the time of analysis. The four most pertinent fertility outcomes are displayed in Figure 1.

### 3.3. Disease Recurrence & Death

**Figure 2:**
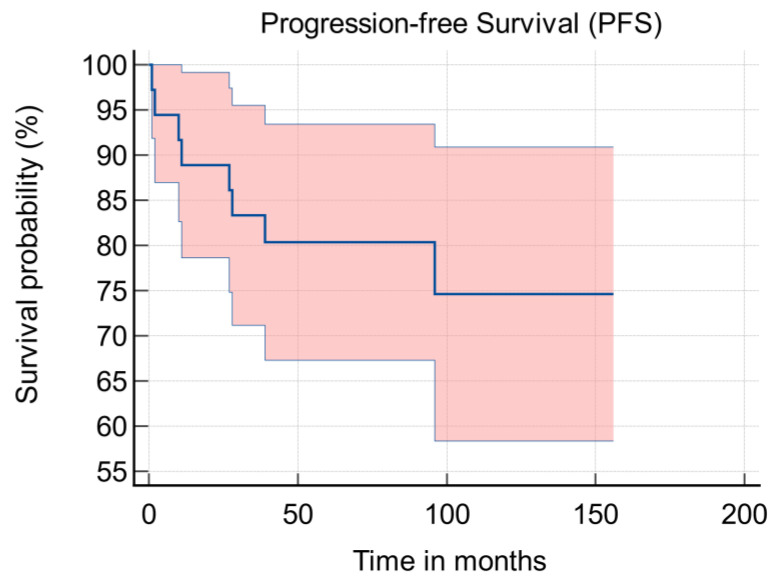
Kaplan-Meier progression free survival graph.

Disease recurrence was detected in eight of 36 patients (22%). Histological subtypes were: EOC (five [63%]); SCST (two [25%]) and GCOT (one [13%]). In six of the eight recurrences, a hysterectomy was performed, one had adjuvant chemotherapy and one declined any further treatment. Out of the eight patients, one had a successful pregnancy prior to hysterectomy. The mean time from surgery to recurrence was 125.3 months (95% CI: 106.5–144.1 months) [Figure 2]. The five year progression-free survival (PFS) was 80% and 10-year survival was 75%.

**Figure 3:**
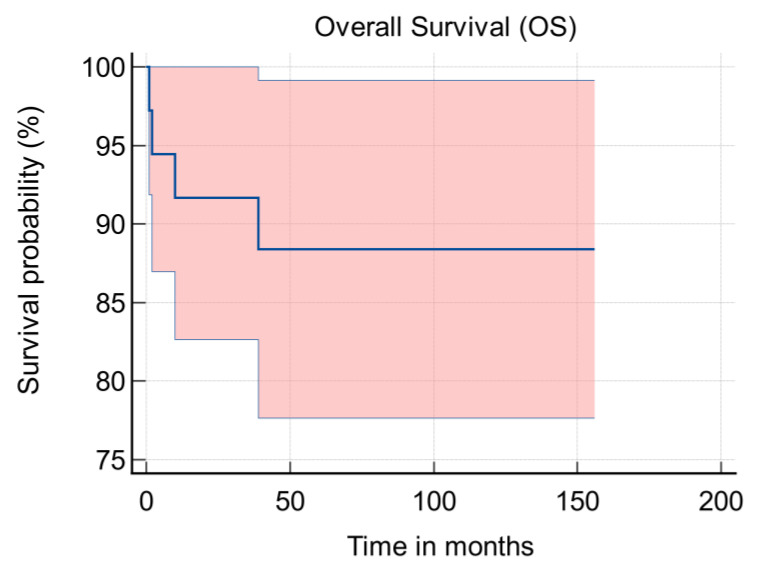
Kaplan-Meier survival graph.

Four patients died from their disease. These cases are summarised in Table 2. The mean time from surgery to death was 139.5 months (95% CI: 124.3 to 154.8 months) [Figure 3]. The five-year overall survival was 88%.

A disease stage of greater than 1A had a significant association with mortality (*p* = 0.02), and recurrence (*p* = 0.07). Similarly, tumour grade greater than 1 was associated with death (*p* = 0.02) but not with recurrence (*p* = 0.2). None of the following factors had a significant association with recurrence or death: histological subtype; open versus laparoscopic surgery; cystectomy versus oophorectomy or positive pregnancy test. There was a significant association between IVF and recurrence (*p* = 0.03), but not death. The findings are summarised in Table 3.

## 4. Discussion

This is one of the largest prospective questionnaire studies to date assessing the reproductive outcomes of women following FSS for ovarian malignancy exclusively. Due to the case mix of central London’s population, the patient demographics were varied, allowing for generalisability of this study. It appears to be the only study of its nature to be UK based, to include women with all histological subtypes of ovarian cancer, women over the age of 40 years as well as to use ART. The inclusion of a number of risk factors for recurrence as well as analysis of pregnancy outcomes should add extra evidence when counselling patients considering FSS. Additionally, the duration of follow up in this study is considerably longer than many others.

The mean age at diagnosis in this study was 30 years. This is half the average age at diagnosis for ovarian cancer [6]. This is due to the fact that FSS is only offered to patients of child-bearing age and usually accepted by women who have not completed their family. Additionally, London hospitals care for a younger demographic than the rest of the UK [12]. While the Royal College of Obstetricians and Gynaecologists advocate that all women with stage 1A EOC should be considered for FSS, Cancer Research UK reports that only 31% of EOCs are stage 1 at diagnosis, with a small proportion being 1A [15,16]. This is reflected in the fact that over a 10-year period the sample size of 47 is relatively small. The distribution of the histological subtypes in this study mimicked that of previous large cohort studies [17].

Just under half (47%) of patients attempted to conceive following FSS. While this number may initially seem low, it is on the higher side compared to other studies which quote rates of 16–50% [18]. There was an overall successful pregnancy rate of 59%, which is comparable to a number of other studies which quote ranges of 54–67% [6,19]. The IVF rate of (12%) was comparable to other studies, but markedly higher than the general population [20,21]. The need for IVF is believed to be due to reduced ovarian reserve and alterations to pelvic anatomy following surgery [21]. In the UK, fertility treatment is available free of charge to women without children who cannot conceive following such treatment. The caesarean section rate in this cohort was comparable to the background rate in this hospital at the time of analysis. There was documentation of an indication for caesarean section that was not related to previous FSS in all cases, making it reasonable to conclude that a history of FSS should not alter the mode of delivery for the majority. The finding of medical complications in 17% of this cohort again was not higher than background rates, which suggests that there is no requirement for increased medical screening [22]. While the preterm birth rate in this study was higher than the UK’s background rate, in two out of the three cases this was due to early delivery for twin pregnancies. While this sample size is small, there is no other evidence to suggest a need for additional preterm birth surveillance in this cohort.

Why 53% of patients chose not to attempt to conceive following FSS is complex, and there are likely multifactorial reasons for this that are beyond the scope of this study. Regardless of fertility outcomes, it is important to note that fertility preservation is one of the major concerns for women under the age of 40 seeking cancer treatment [16]. When cancer treatment results in permanent infertility, it causes great emotional distress, and this is one of the major issues affecting the patient’s long-term quality of life [23].

The miscarriage rate of 38% was higher than expected compared to the national rate of 18% for this age group [24]. One systematic review of reproductive outcomes following FSS showed an average miscarriage rate of 15% [25]. This finding cannot be explained. Out of the six patients who experienced pregnancy losses, two (33.3%) experienced three or more miscarriages. While investigations for recurrent pregnancy loss were normal, this sample size is too small to draw any conclusion on the risk of miscarriage following FSS.

The recurrence rate in this study was 22%, which is slightly higher than other studies which quote figures between 13 and 15%, [6,7,26] This may be explained by the fact that this study included patients over the age of 40 and women with higher grade tumours. The finding that death was associated with a disease stage greater than 1A supports large systematic reviews [10]. The finding that higher grade tumours had a positive association with death is also supported by previous studies which suggest that patients with high grade tumours should be counselled about the benefit of full staging surgery either immediately or as soon as their family is complete [6,26]. The five-year and 10-year PFS rates in this study were both 5% lower than rates quoted in a recent systematic review [10]. This may partly be explained by this study having a higher proportion of disease stage > 1A and tumour grades of two or three opting for FSS. The finding of an association between the use of IVF and recurrence is of interest. At least one case-control study and a number of case reports have suggested a causal relationship between ART and invasive EOC [27]. This theory has been refuted in a number of small prospective trials, and due to the small number of IVF treatments in this study, no meaningful relationship can be determined between IVF and risk of recurrence or death [28].

Limitations of this study are the fact that it used retrospectively collected data and relied on the patient's memory spanning up to 10 years, and was single-centred. As a significant proportion of this cohort were in their 20s and 30s, and some surgeries were performed within the preceding five years, therefore attempts at conception may occur in the future. For this reason the cohort will be re-analysed in five to 10 years’ time. While this study did not find a significant difference in outcomes based on histological subtypes, the sample size for less common types such as yolk sac tumours was small (*n* = 2), therefore it is not possible to draw definite conclusions on this.

Future work in this field requires a large prospective, multi-centre study assessing the long term outcomes for more advanced grades of tumours and rarer histopathological subtypes. Further work is required in assessing the complex reasons why women may not choose to attempt to conceive following fertility sparing surgery, exploring both physical and psychological barriers.

## 5. Conclusions

This study suggests that while a large proportion of women will not attempt to conceive following FSS, for those who do, the likelihood of achieving an uncomplicated live birth is high. Careful counselling is required when considering the type of surgery to be performed based on the stage of disease and tumour grade.

## Figures and Tables

**Table 1:** Patient and tumour demographics.

Demographic Parameter	Quantitive or Qualititive Diescription of the Demography				
**Age**	*30.2 years (95% CI: 27.6 to 33.0 years)*				
**Ethnicity**	*Caucasian: 21/36 (58.3%)*	*Black: 9/36 (25%);*	*Middle Eastern: 3/36 (8.3%)*	*South Asian: 2/36 (5.6%)*	*South East Asian: 1/36 (2.8%).*
**Comorbidities**	*PCOS & endometrial hyperplasia: 1/36 (2.8%)*	*Asthma: 1/36 (2.8%)*	*Obesity: 1/36 (2.8%)*		
**Treatment**	*USO: 32/36 (88.9%)*	* Cystectomy: 4/36 (11.1%) *			
**Disease Stage**	*1A: 27/36 (75%)*	*1C: 5/36 (13.8%)*	*2A: 1/36 (2.8%)*	*2C: 1/36 (2.8%)*	*3C: 2/36 (5.6%)*
**Tumour Grade**	*1: 22/36 (61%)*	*2: 5/36 (14%)*	*3: 9/36 (25%).*		
**Histology (Epithelial Ovarian Tumours)**	*mucinous cell adenocarcinoma: 7/36 (19.4%)*	*Clear cell carcinoma: 4/36 (11.1%)*	*serous cell adenocarcinomas: 2/36 (5.6%)*	*undifferentiated carcinoma: 1/36 (2.8%)*	*poorly differentiated squamous cell carcinoma: 1/36 (2.8%)*
**Histology (Sex Cord Stromal Tumours)**	*Adult Granulosa cell tumours: 12/36 (33.3%)*	*Sclerosing Stromal tumour: 1/36 (2.8%)*			
**Histology (Germ Cell Ovarian Tumours)**	*Immature teratoma: 5/35 (14.3%)*	*Yolk sac germ cell tumours: 2/36 (5.6%)*	*Dysgerminoma: 1/36 (2.8%)*	*mixed germ cell tumour: 1/36 (2.8%)*	

**Table 2:** Summary of deceased patients.

Patient	Age at Diagnosis	Type of Surgery	Histology	Grade	Stage	Time to Recurrence (Months)	Further Treatment	Time to Death (Months)
1	35	USO, Bilateral Pelvic Lymph Node dissection (BPLND), Para-Aortic Lymph Node Dissection (PALND), appendicectomy & omentectomy	Clear cell carcinoma	3	1C	9	Total abdominal hysterectomy (TAH, Bilateral salpingo oophorectomy (BSO), bowel resection & diaphragmatic stripping. 6 cycles of adjuvant chemotherapy & 5 cycles of palliative chemotherapy.	31
2	39	USO & omental biopsies	Squamous cell carcinoma	3	1C	1	Nil	1
3	40	USO & BPLND	Immature teratoma	3	2A	3	Palliative debulking surgery	8
4	25	USO, BPLND, PALND & omentectomy.	Mucinous carcinoma	2	1A	35	3 cycles of chemotherapy. Radiotherapy to groin mass.	57

**Table 3:** Summary of potential risk factors and association with recurrence and death.

	Association with Recurrence (*p* Value for Chi-Square Test)	Association with Death (*p* Value for Chi-Square Test)
Disease stage > 1A	*p* = 0.07	*p* = 0.02
Tumour grade > 1	*p* = 0.2	*p* = 0.02
In vitro fertilisation	*p* = 0.03	NA (No deaths in pregnancy group)
Histological subtype	*p* = 0.4	*p* = 0.3
Open versus laparoscopic	*p* = 0.5	*p* = 0.3
Cystectomy versus oophorectomy	*p* = 0.2	*p* = 0.5
Pregnancy	*p* = 0.2	*p* = 0.2

## Data Availability

Original data can be made available on request to the corresponding author.

## Appendix A. Study Questionnaire

1. Have you tried to fall pregnant since your surgery: YES NO
2. Have you had a positive pregnancy test YES NO
3. What was the outcome of each of your pregnancies Child Loss
4. Number of each ____ _____
5. If did try to conceive, was it natural or ART Natural ART
6. If couldn’t fall pregnant was this investigated YES NO
Abnormality detected ______________________________________________________
7. If did fall pregnant, any complications? YES NO
Details ________________________________________________________________
8. If did fall pregnant, mode of delivery? Vaginal C-Section
9. Any disease recurrence or further treatment YES NO
